# Development of an X-ray ionization beam position monitor for PAL-XFEL soft X-rays

**DOI:** 10.1107/S1600577524006003

**Published:** 2024-07-29

**Authors:** Seonghan Kim, SunMin Hwang, Hoyoung Jang, Seungcheol Lee, HyoJung Hyun

**Affiliations:** a Pohang Accelerator Laboratory, POSTECH, Pohang, Gyeongbuk37673, Republic of Korea; Deutsches Elektronen-Synchrotron, Germany

**Keywords:** X-ray free-electron laser, soft X-rays, ionization, *in situ* diagnostics, beam position monitoring

## Abstract

The Pohang Accelerator Laboratory X-ray Free-Electron Laser (PAL-XFEL) operates hard X-ray and soft X-ray beamlines for conducting scientific experiments providing intense ultrashort X-ray pulses based on the self-amplified spontaneous emission (SASE) process. The X-ray free-electron laser is characterized by strong pulse-to-pulse fluctuations resulting from the SASE process. Therefore, online photon diagnostics are very important for rigorous measurements. The concept of photo-absorption and emission using solid materials is seldom considered in soft X-ray beamline diagnostics. Instead, gas monitoring detectors, which utilize the photo-ionization of noble gas, are employed for monitoring the beam intensity. To track the beam position at the soft X-ray beamline in addition to those intensity monitors, an X-ray ionization beam position monitor (XIBPM) has been developed and characterized at the soft X-ray beamline of PAL-XFEL. The XIBPM utilizes ionization of either the residual gas in an ultra-high-vacuum environment or injected krypton gas, along with a microchannel plate with phosphor. The XIBPM was tested separately for monitoring horizontal and vertical beam positions, confirming the feasibility of tracking relative changes in beam position both on average and down to single-shot measurements. This paper presents the basic structure and test results of the newly developed non-invasive XIBPM.

## Abstract 2

An X-ray ionization beam position monitor developed for PAL-XFEL soft X-rays is introduced.

## 1. Introduction

The Pohang Accelerator Laboratory X-ray Free-Electron Laser (PAL-XFEL) (Ko *et al.*, 2017 ▸; Kang *et al.*, 2017 ▸) has provided intense, ultrashort and coherent X-ray pulses based on the self-amplified spontaneous emission (SASE) process (Kondratenko & Saldin, 1980 ▸; Bonifacio *et al.*, 1984 ▸) for a number of scientific experiments, in particular ultrafast science. The PAL-XFEL consists of hard X-ray and soft X-ray beamlines that provide photons with energy ranging from 2 to 15 keV and from 0.25 to 1.25 keV, respectively. In the case of the hard X-ray beamline, two experimental hutches, *i.e.*X-ray Scattering and Spectroscopy (XSS) (Park, Eom *et al.*, 2016 ▸) and Nano Crystallography and Coherent Imaging (NCI) (Park, Kim *et al.*, 2016 ▸; Sung *et al.*, 2021 ▸), have a tandem geometry and share the undulator line and optics hutch. Similar to the hard X-ray beamline, there are two endstations at the soft X-ray beamline. These endstations are Resonant Soft X-ray Scattering (RSXS) (Jang *et al.*, 2020 ▸) and X-ray Emission/Absorption Spectroscopy (XES/XAS) (Park *et al.*, 2018 ▸). The X-ray free-electron laser (FEL) is characterized by strong pulse-to-pulse fluctuations on the intensity, position, spectrum and photon energy resulting from the SASE process (Saldin *et al.*, 1998 ▸, 2010 ▸). Therefore, online photon diagnostics are very important for the experiments. The quadrant beam position monitor (QBPM) is used for monitoring the beam position and pulse energy of the XFEL at the hard X-ray beamline. The QBPM, obtained from FMB Oxford, UK, consists of four photodiodes (S-100VL from OSI Optoelectronics) that detect backscattered X-rays from a thin film placed in the beam path (Alkire *et al.*, 2000 ▸; Feng *et al.*, 2011 ▸; Tono *et al.*, 2011 ▸). The concept of the QBPM is not valid for soft X-rays due to strong attenuation at the thin films. Therefore, a gas monitor detector (GMD) based on the photo-ionization of noble gases (Tiedtke *et al.*, 2008 ▸, 2014 ▸) has been utilized only for pulse energy monitoring and signal normalization at the soft X-ray beamline. To track the beam position, we developed a prototype of an X-ray ionization beam position monitor (XIBPM). The XIBPM utilizes the ionization of either the residual gas in a vacuum (Sachwitz *et al.*, 2008 ▸; Miessner *et al.*, 2011 ▸) or introduced krypton gas to monitor changes in beam position at the soft X-ray beamline of the PAL-XFEL. This paper presents the design, manufacture and assembly of the XIBPM, and describes test results under various conditions, including beam energy in both monochromatic and pink beam modes, applied electric field and beam position.

## 2. X-ray ionization beam position monitor

**Figure 1:**
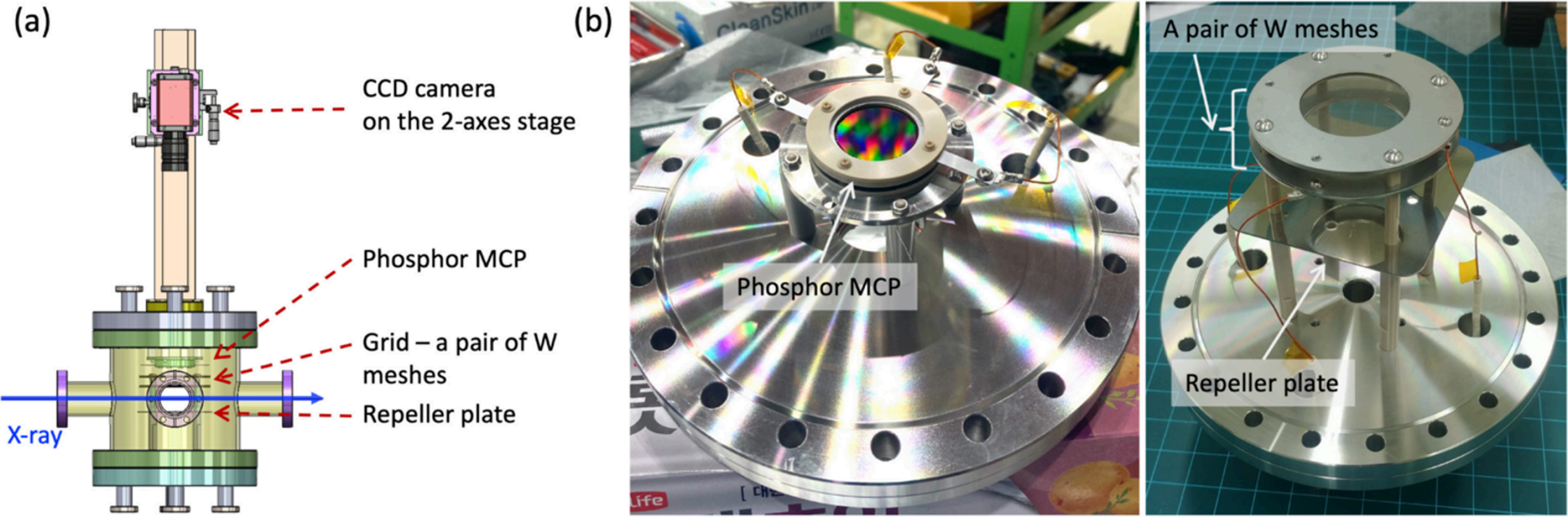
(*a*) Design of the XIBPM. To simultaneously measure beam profiles in the horizontal and vertical directions, a pair of XIBPMs is required, positioned with a 90° rotation between them. (*b*) Manufacturing and assembly of the XIBPM. The MCP with a phosphor screen was assembled on one side, while a pair of tungsten meshes and the repeller plate were assembled on the other side.

Fig. 1 ▸ shows a schematic drawing, and manufacturing and assembly of the XIBPM. The XIBPM consists of two CF flanges each with a 10-inch outside diameter; one is for the detector and the other is for the creation of the electric field. The detector is composed of the microchannel plate (MCP) with a phosphor screen (F2223-21P with P43, Hamamatsu Photonics) and out-vacuum CCD camera (Manta G-046B, Allied Vision). The effective areas of the MCP and the screen are 27 mm and 24 mm in diameter, respectively. The channel diameter of the MCP was 12 µm, and the grain size of the screen was 2–3 µm with a decay time of 3.2 ms from 100% to 1% (Stanford Computer Optics, 2024 ▸; Photonis, 2024 ▸). The CCD camera, with an 8.3 µm × 8.3 µm pixel size and a 780 × 580 resolution, was installed on the two-axes stage with a travel length of ±6.5 mm, and the stage was assembled to the guide rail. The system for the creation of the electric field consisted of a repeller plate for pushing the photo-ions and a grid with a pair of tungsten meshes for creating the equi­potential. The repeller plate was composed of stainless steel with a thickness of 1 mm. The plate had a rectangular shape with rounded corners. Each tungsten mesh was held by two rings made of stainless steel with a thickness of 1 mm and an inner diameter of 45 mm. The aperture size and wire diameter of the tungsten mesh were 0.15 and 0.02 mm, respectively.

**Figure 2:**
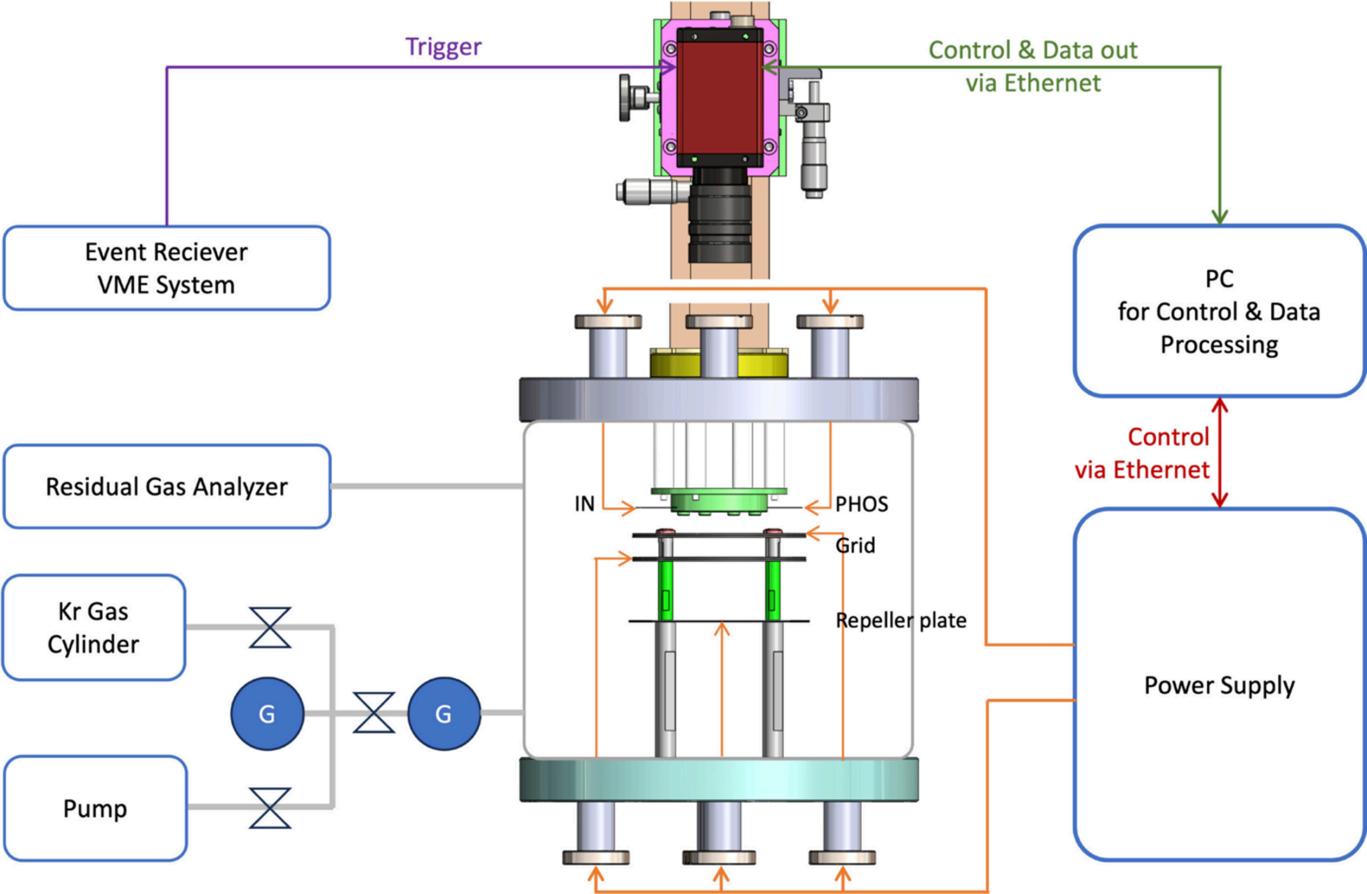
Schematic diagram of the XIBPM. The voltages for the tungsten meshes, repeller plate and MCP with a phosphor screen were supplied via SHV feedthroughs from the power supply. The beam trajectory was recorded by an out-vacuum CCD camera operated using an external trigger from the event receiver VME system. Additionally, a system for Kr gas injection was integrated into the XIBPM chamber.

The created photo-ions moved along the path of the electric field that was generated by applying a voltage to the repeller plate and grid. The photo-ions were amplified by the MCP, and their footprints were subsequently recorded using the phosphor screen and CCD camera. The MCP features three leads, namely IN, OUT and PHOS. To facilitate ion detection, it is essential to ground the OUT lead, while adjusting the gain can be achieved by modifying the voltage applied to the IN lead. Applying a positive voltage to the PHOS lead enables the electrons amplified by the MCP to create a footprint on the phosphor screen. The CCD camera operated in synchronization with an external trigger from the PAL-XFEL event receiver VME system. Additionally, a system for the Kr gas injection was assembled in the XIBPM chamber together with a residual gas analyzer. A schematic diagram of the XIBPM is shown in Fig. 2 ▸.

To monitor the beam position in both directions, *i.e.* horizontal and vertical, a pair of the XIBPMs is required. The structures of both the XIBPMs can be exactly the same. However, one of them should be installed with a 90° rotation on the *Z*-axis. One XIBPM was manufactured, assembled and subsequently installed at the soft X-ray beamline of PAL-XFEL. To test the XIBPM under various beam conditions, it was positioned downstream of the monochromator and upstream of the exit slit. Depending on the direction of the beam position change being tested, the XIBPM’s installation varied.

## 3. Tests with soft X-ray pulses

### 3.1. Experimental procedure

The XIBPM was tested under various conditions. Monochromatic and pink beams (mono-beam mode and pink-beam mode) with energies of 500, 700, 900 and 1100 eV were used. The photo-ion signals were initially obtained using only the residual gas in the vacuum (residual gas mode) to check the feasibility of avoiding a bulky differential pumping system. Subsequently, krypton gas (Kr gas mode) was introduced to increase the signal-to-noise ratio and for comparison. The vacuum pressures for the residual gas mode and the Kr gas mode were maintained at ∼10^−8^ and ∼5 × 10^−7^ Torr, respectively. The vacuum status was checked by using the residual gas analyzer before and after the injection of Kr gas. When the XIBPM operated in the residual gas mode, the highest peak was observed for H_2_ at a pressure of 2 × 10^−8^ Torr, followed by H_2_O at 1 × 10^−8^ Torr and N_2_ at 2.5 × 10^−9^ Torr. In the Kr gas mode, the Kr peak appeared to be comparable with the H_2_O peak. The voltage for the phosphor screen of the MCP was fixed to be +3000 V, and the gain of the MCP was adjusted with the application of the voltages to the IN lead of the MCP. The applied voltages varied depending on the beam condition and gas mode. The voltage of the repeller plate was also varied depending on the test subject and environments, and it was chosen to be in the range +2000 to +4000 V. Therefore, the electric fields determined by the potential difference between the repeller plate and the MCP IN plate were changed. The voltages for each mesh in the grid were also adjusted in accordance with the electric field. The voltages decreased linearly with the distance between the repeller plate and the MCP IN plate.

**Figure 3:**
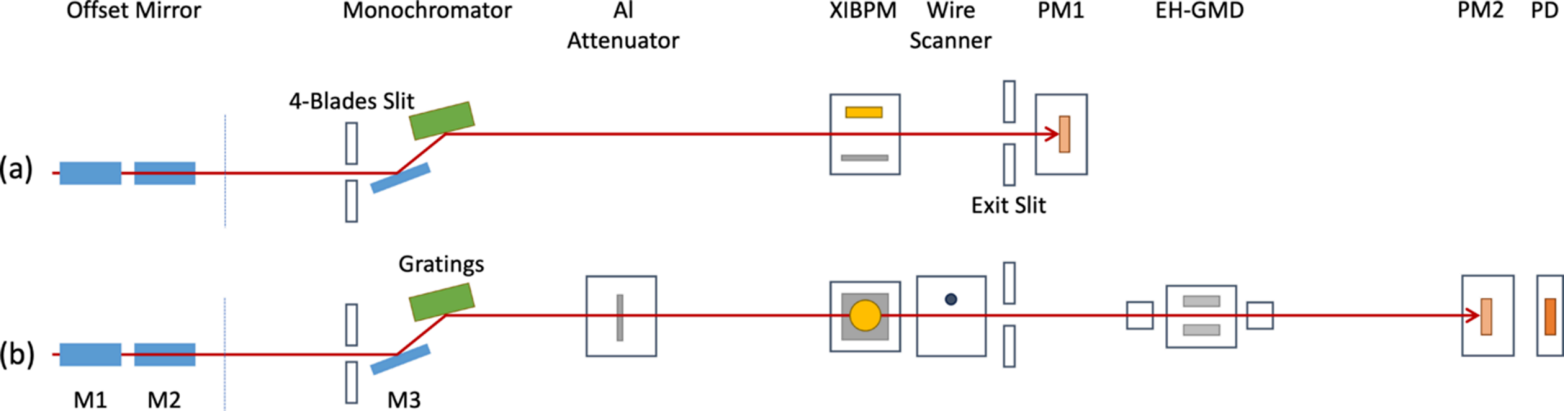
Test configurations are shown in a side view for (*a*) measuring horizontal beam position changes and (*b*) measuring vertical beam position changes. The XIBPM was positioned downstream of the monochromator and upstream of the wire scanner, installed with a 90° rotation according to the test configuration.

The test configurations, depending on the installation of the XIBPM, are depicted in Fig. 3 ▸. To mimic the horizontal beam position change, the beam was cut by changing the position of the four-blades slit in front of the monochromator in mono-beam mode. For changing the beam position in the vertical direction, the path of the beam itself was manipulated by adjusting the angle of the M3 mirror inside the monochromator in pink-beam mode. The 2-D images of the XIBPM and the pop-in monitor (PM) with the Ce:YAG screen and CCD camera were recorded together for each setting. Two PMs, *i.e.* PM1 and PM2, were used for testing. The PM1 was located between the exit slit and the GMD at the experimental hall (EH-GMD). Since PM2 was newly installed downstream of the RSXS endstation, PM1 was removed. The wire scanner, with tungsten wires of 100, 200 and 500 µm diameters, was installed along with the new PM2. It was positioned just before the exit slit, which is located at the vertical focal plane created by the M3 mirror. The X-ray beam flux was modulated by varying the thickness of the aluminium attenuator to assess the linearity of the XIBPM. The thickness values were 0 (bypass), 300, 600, 1200 and 2400 nm.

**Figure 4:**
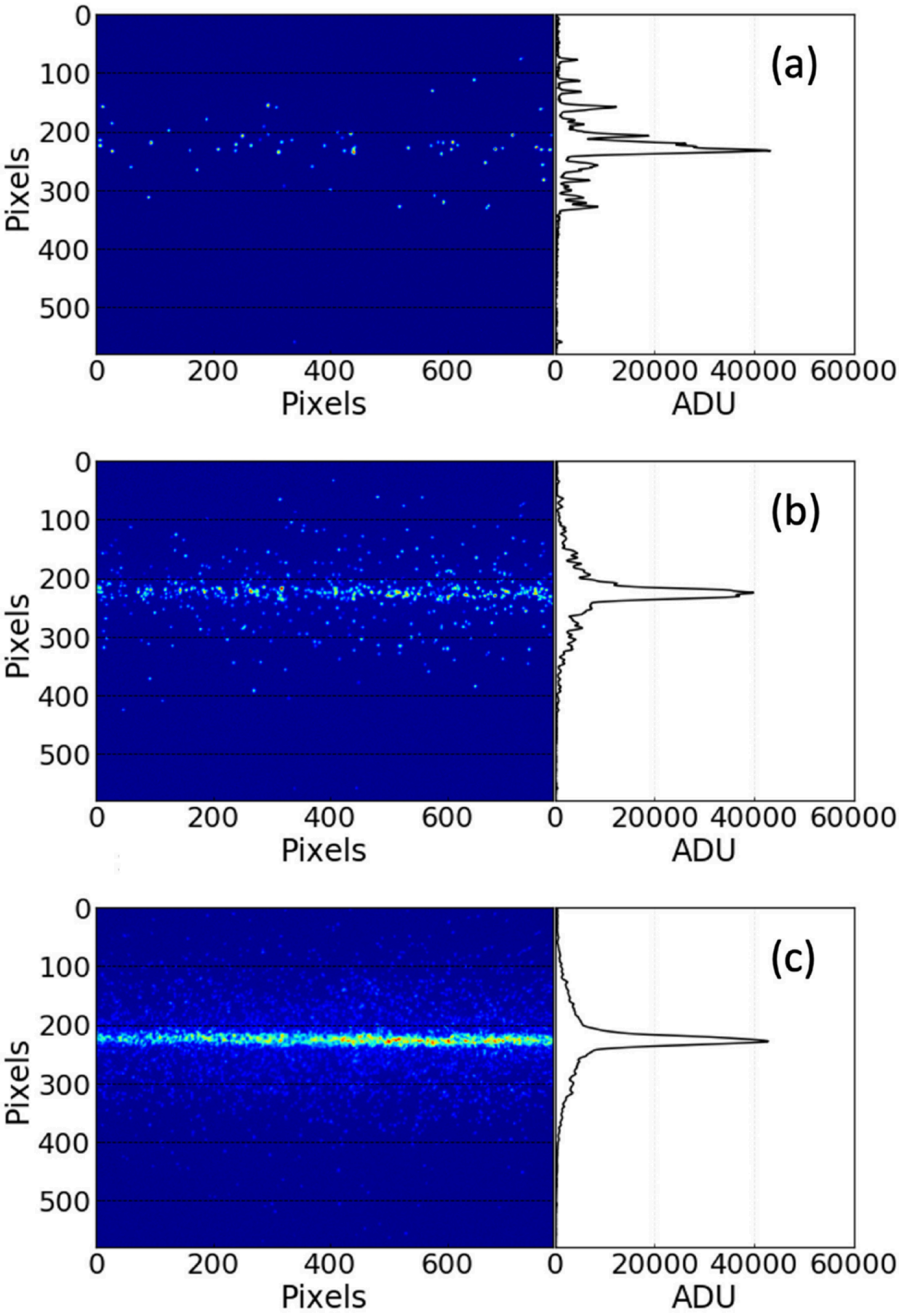
Trajectory and profile of the beam at a mono-beam energy of 500 eV with a 1 mm exit slit in the residual gas mode and an electric field of 885 V cm^−1^. The trajectory on the 2D image and the profile were obtained by accumulating and averaging (*a*) a single X-ray pulse, (*b*) ten X-ray pulses and (*c*) 100 X-ray pulses.

After background subtraction, the image data from the XIBPM and PMs were projected pulse by pulse, in grouped pulses, or as the total number of pulses. The subsequent analysis involved determining the maximum height and identifying the pixel positions corresponding to the maximum height. The widths of the profiles were determined through fitting with the Gaussian function. Depending on the circumstances, the waveforms of the photo-ions and photo-electrons from the EH-GMD and the photo-diode (PD) downstream of PM2 were also saved together. The charges of the EH-GMD and PD were obtained by integrating the waveforms. Fig. 4 ▸ shows an example of the image data and profile of the XIBPM at a mono-beam of 500 eV and the residual gas mode under an electric field of 885 V cm^−1^, depending on the number of X-ray pulses that were accumulated and averaged. The trajectory of the beam and profile displayed in analog-to-digital units (ADU) were clearly visible for a larger number of pulses. The signal-to-noise ratio for a single pulse was estimated to be 2.92 ± 0.26 on average for each pixel.

The XIBPM and PMs were calibrated using images of a grid pattern obtained at the corresponding CCD camera settings for each instrument. They were estimated to be 29.4 ± 0.4, 28.0 ± 0.5 and 56.5 ± 0.5 µm pixel^−1^ for the XIBPM, PM1 and PM2, respectively.

### 3.2. Results and discussions

#### 3.2.1. Amplitude, width and peak position of profiles

**Figure 5:**
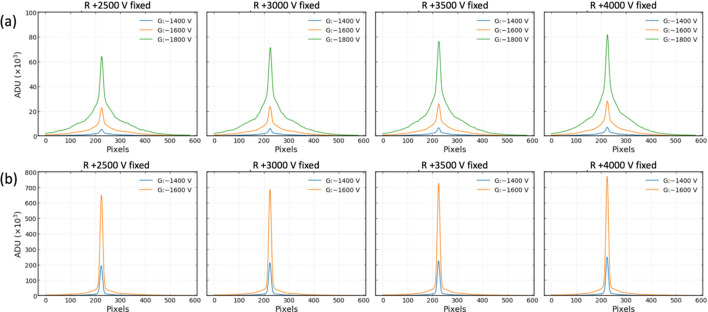
Beam profiles at 900 eV of mono-beam mode. The voltages for the repeller plate (R) and MCP gain (G) of the XIBPM were adjusted for (*a*) the residual gas mode and (*b*) the Kr gas mode. The profiles were averaged over 1200 X-ray pulses for each setting.

**Figure 6:**
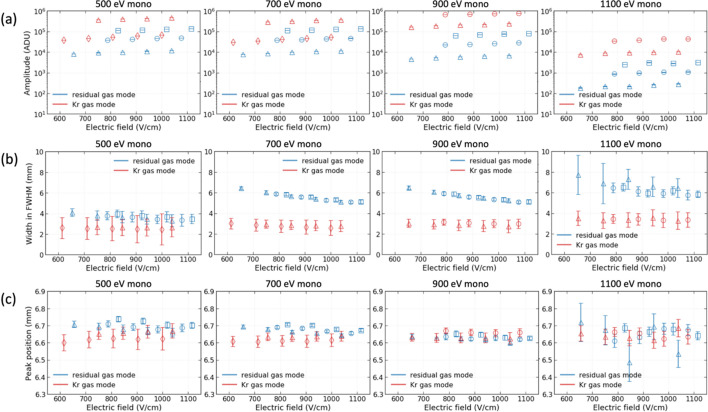
The profiles were obtained from the XIBPM by adjusting the electric field and X-ray energy in mono-beam mode, either in the residual gas mode or the Kr gas mode. They were averaged over 1200 X-ray pulses for each setting and fitted using a triple Gaussian function. (*a*) Maximum amplitude, (*b*) width and (*c*) peak position of profiles are shown as functions of the electric field. Thin-diamond, triangle, circle and square symbols represent MCP gains of −1200, −1400, −1600 and −1800 V, respectively.

The basic properties, namely amplitude, width and peak position, of the profiles were evaluated in mono-beam mode by varying the beam energy and electric field for both the residual gas and Kr gas modes within the test configuration shown in Fig. 3 ▸(*a*). In the residual gas mode, voltages of −1400, −1600 and −1800 V were applied to change the MCP gain, irrespective of the beam energy. Voltages of −1200 and −1400 V (−1400 and −1600 V) were applied for the beam energies of 500 and 700 eV (900 and 1100 eV) in the Kr gas mode. The voltage of the repeller plate was changed from +2000 to +4000 in steps of 500 V. A total of 1200 X-ray pulses were collected for each setting at a repetition rate of 60 Hz. The profiles obtained by averaging with the total number of X-ray pulses were fitted by using the triple Gaussian function. Fig. 5 ▸ presents the averaged profiles at 900 eV depending on the voltages of the repeller plate and the MCP gain for both gas modes as an example. Fig. 6 ▸ illustrates the distributions of amplitude, width and peak position of the profiles as a function of the electric field, depending on the beam energy and operation gas mode.

The amplitudes increased gradually depending on the voltage of the repeller plate when the MCP gains were the same. On the other hand, the amplitudes showed a large increase depending on the gain of the MCP when the voltages of the repeller plate were the same (see Fig. 5 ▸). Therefore, the data points could be grouped horizontally depending on the voltage of the MCP gain [see Fig. 6 ▸(*a*)]. By injecting Kr gas, the amplitude increased by approximately dozens of times at the same MCP gain. The distributions of the width were very similar at 700 and 900 eV. However, overall the width increased as the beam energy increased for both the residual gas and Kr gas modes [see Fig. 6 ▸(*b*)]. After the residual gas or Kr gas was ionized by X-ray pulses, the remaining energy was converted into the kinetic energy of the photo-electrons and photo-ions. Due to the significantly larger mass of photo-ions, most of the kinetic energy was taken up by the photo-electrons. Despite the potential negligibility of photo-ion kinetic energy, it increased proportionally with photon energy. Consequently, it was speculated that photo-ions with higher kinetic energy may experience diminished influence from the electric field, potentially resulting in a broader profile width. Conversely, a stronger electric field narrowed the width. The width of the profile clearly decreased with increasing electric field in the residual gas mode; however, this feature dis­appeared in the Kr gas mode for all the beam energies. Excluding the results at the energy of 1100 eV, it was observed that the errors of the width in the Kr gas mode were larger than those of the residual gas mode. Contrary to our expectations, the effect of the electric field on the peak position was observed consistently across the remaining energies, except for 1100 eV [see Fig. 6 ▸(*c*)]. In contrast to the amplitude, increasing the voltage for the repeller plate slightly decreased the peak position at the same MCP gain. This effect was clearly demonstrated in the residual gas mode, while, in the Kr gas mode, increasing the voltage for the repeller plate had no impact on the peak position. Additionally, at the same repeller plate voltage, increasing the MCP gain resulted in a higher peak position in both residual gas and Kr gas modes, which may be attributed to the imbalance in the electric field caused by the 20 mm length of the MCP IN lead, as shown in Fig. 1 ▸(*b*). The effects on the width and peak position due to variations in the beam energy and electric field appeared more pronounced in the residual gas mode, possibly indicating the influence of the kinetic energy of residual gas photo-ions and Kr ions resulting from the mass difference. The larger errors revealed in the width and peak position at the residual gas mode of 1100 eV were because of the poor signal-to-noise ratio, which was demonstrated at a low MCP gain.

During the development of the XIBPM, it was revealed that vacuum quality control, namely chamber bake-out and gas pipe flushing, is very important. It was observed that the width under the electric field of 846 V cm^−1^ was improved by approximately 12% in the residual gas mode after the bake-out and flushing. Subsequently, the width under the same electric field and the Kr gas mode was smaller by approximately 48% than that in the residual gas mode.

The profile widths obtained in pink-beam mode within the test configuration shown in Fig. 3 ▸(*b*) were compared with the vertical beam size measured by the knife-edge method using the 500 µm-diameter tungsten wire in the wire scanner and the EH-GMD. The MCP gain voltages of the XIBPM were adjusted to be −1400, −1600 and −1800 V (−1400 and −1600 V), which were applied for the overall beam energy under the residual gas mode (Kr gas mode). The measured vertical beam sizes using the wire scanner were 63.5 ± 1.6, 59.2 ± 1.8, 63.3 ± 2.9 and 57.9 ± 7.5 µm full width at half-maximum (FWHM) for 500, 700, 900 and 1100 eV, respectively. The XFEL beam was focused vertically around the exit slit, and thereafter started to be diverse. Thus, the measured profile widths using PM2 were 637.7 ± 5.1, 602.4 ± 4.3, 533.9 ± 3.2 and 471.4 ± 2.3 µm FWHM for 500, 700, 900 and 1100 eV, respectively. These observations were consistent results within errors of 3σ, considering the distances of the devices installed from the M3 mirror of the monochromator. However, the profile widths of the XIBPM were about six to nine times larger than the beam size measured by the wire scanner, even though the XIBPM and the wire scanner were located closely. As observed in the peak position changes depending on the MCP gain voltage, the strong electric field around the MCP IN lead may also cause a bending of the trajectory of the photo-ions, resulting in a broader profile.

#### 3.2.2. Displacements

**Figure 7:**
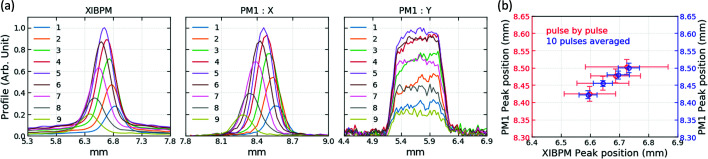
(*a*) Profiles and (*b*) displacements of the XIBPM and PM1 at 500 eV in mono-beam mode. The repeller plate and the MCP gain voltages of the XIBPM were set to be +3000 V and −1600 V, respectively, with the XIBPM operating in the residual gas mode. In (*b*), excluding data points with large error bars in the pulse-by-pulse analysis, only data sets numbered 3, 4, 5 and 6 are presented.

The beam positions were changed artificially to investigate the change of the beam trajectory by using the XIBPM. First, the beam position in the horizontal direction was changed in mono-beam mode by using the four-blade slit, which was located upstream of the monochromator chamber [see Fig. 3 ▸(*a*)]. The four-blade slit motor was adjusted at a total of nine positions, and 600 X-ray pulses were acquired for each position. The exit slit was opened by 1 mm, vertically, and the PM1 was located downstream of the exit slit. Therefore, the Y-profile width of the PM1 was limited by the size of the exit slit. Furthermore, using the four-blade slit mimics the change in the beam position by cutting the beam. Therefore, the amplitude of the profiles was also changed depending on the beam cutting position. The displacements were compared using the peak positions of the XIBPM profile and the PM1 X-profile. The beam energy was 500 eV in the residual gas mode, and was varied from 500 to 1100 eV in steps of 200 eV in the Kr gas mode. The electric field was adjusted to be 846, 885 and 923 V cm^−1^ by changing the MCP gain voltage in the residual gas mode, and was fixed to be 846 V cm^−1^ for the Kr gas mode. Figs. 7 ▸(*a*) and 7 ▸(*b*) show the averaged profile distributions of the XIBPM and the PM1, as well as their displacements in mono-beam mode of 500 eV and the residual gas mode, respectively. Each profile was normalized by the profile that had the maximum height. As shown in Fig. 7 ▸(*b*), the pulse-by-pulse analysis exhibited huge error bars in all the data sets of the XIBPM, since the four-blade slit had cut the beam itself. Averaging at a group of every ten pulses showed a better linear correlation, except at the energy value of 1100 eV for the Kr gas mode, which had a poor signal-to-noise ratio. Using the four-blade slit involved changing the source position as well; therefore, their slopes were compared with the ratio of the distances at which both devices were installed from the horizontal focal plane created by the M2 mirror. The relative percentage error of the displacement ratio for the average of a group of every ten pulses was below 12%, except for 1100 eV. For 1100 eV, the error was approximately 7% when partial data points were used, as shown in Fig. 7 ▸(*b*).

**Figure 8:**
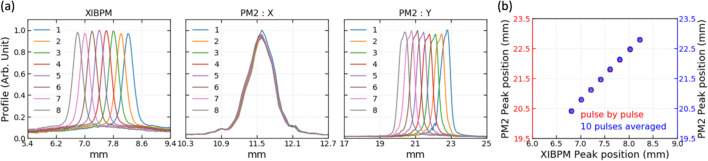
(*a*) Profiles and (*b*) displacements of the XIBPM and PM2 at 500 eV in pink-beam mode. The repeller plate and the MCP gain voltages of the XIBPM were set to be +3000 V and −1600 V, respectively, with the XIBPM operating in the residual gas mode.

**Figure 9:**
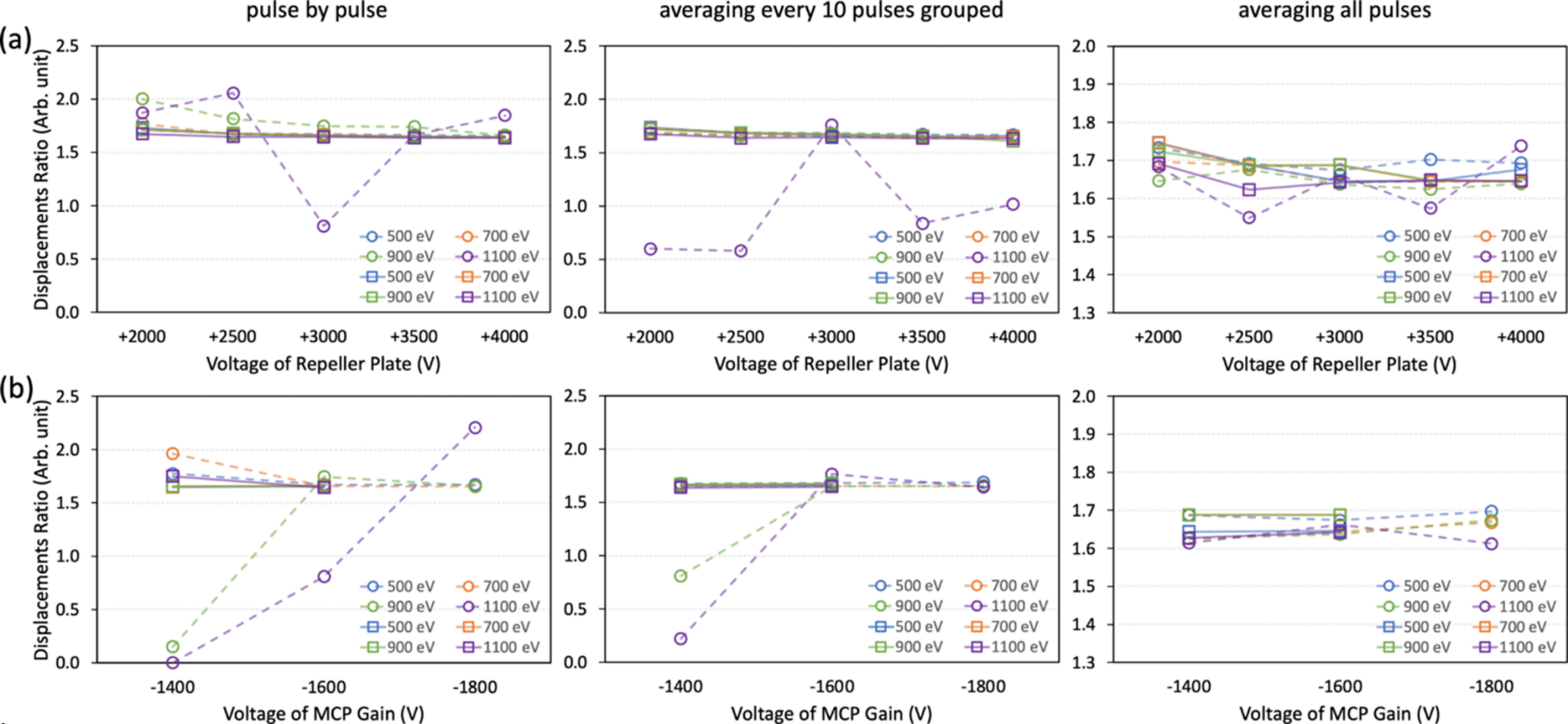
The displacement ratio in pink-beam mode with energies of 500, 700, 900 and 1100 eV was analyzed based on voltages for (*a*) the repeller plate at a fixed MCP gain of −1600 V and (*b*) the MCP gain with a fixed repeller plate voltage of +3000 V of the XIBPM. Results are presented for (left) pulse-by-pulse analysis, (middle) the average of every ten pulses, and (right) the average across all pulses. Open circles represent the residual gas mode, while open squares denote the Kr gas mode.

**Figure 10:**
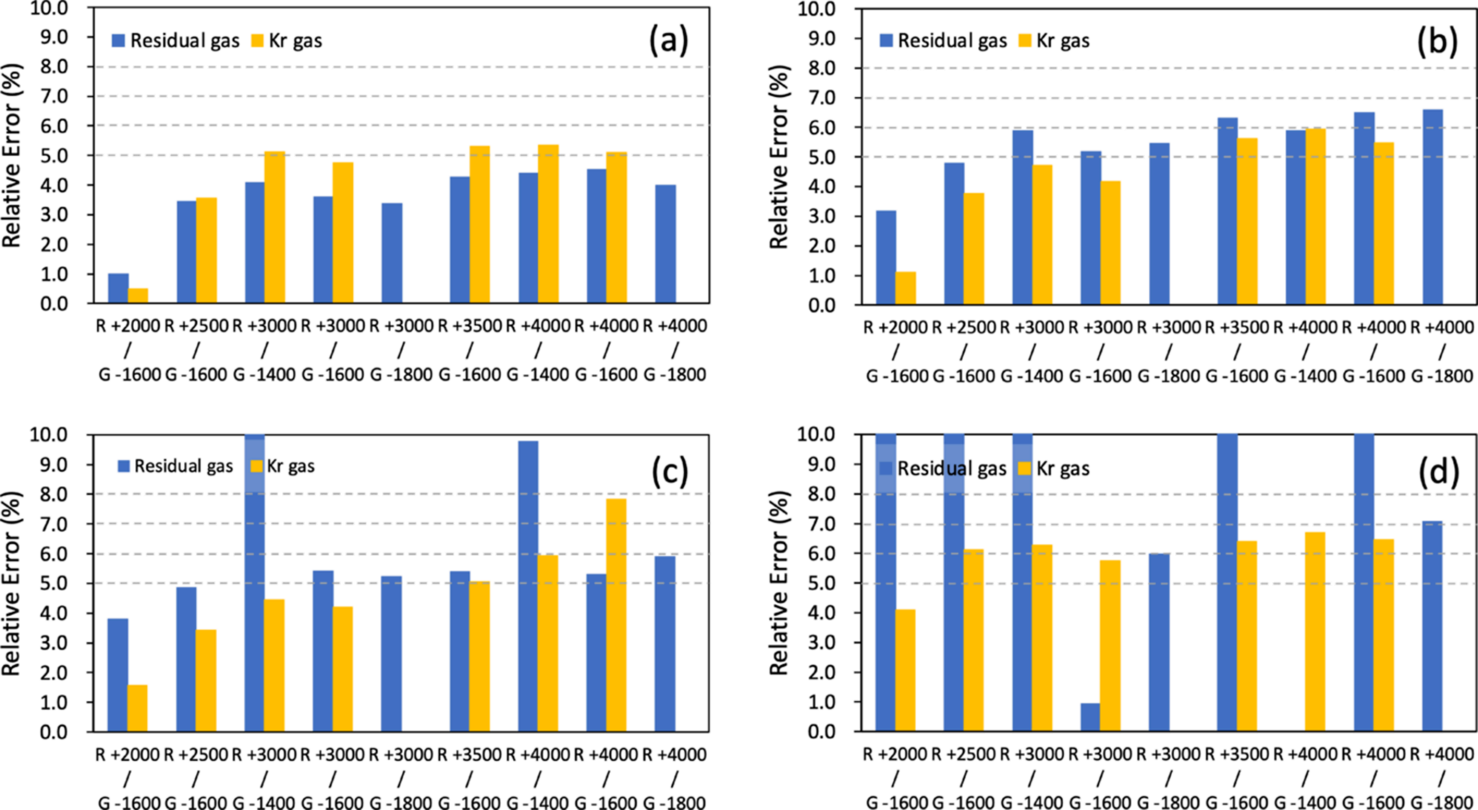
The relative percentage errors of the displacement ratios in pink-beam mode at energies of (*a*) 500 eV, (*b*) 700 eV, (*c*) 900 eV and (*d*) 1100 eV are shown as a function of the voltages for the repeller plate (R) and the MCP gain (G) of the XIBPM. The results are based on the average of every ten pulses.

Secondly, the beam position in the vertical direction was changed in pink-beam mode by using the M3 mirror inside the monochromator chamber [see Fig. 3 ▸(*b*)]. The M3 mirror position was changed to a total of eight positions, and 600 X-ray pulses were acquired for each position. To monitor the change in the beam position in the vertical direction, the XIBPM was rotated by 90° in the *Z*-axis. Beams with energies of 500, 700, 900 and 1100 eV were used for both residual gas and Kr gas modes. The voltages for the repeller plate were varied from +2000 to +4000 V in 500 V increments. The MCP gain voltage was mainly set to be −1600 V, regardless of all the setting voltages of the repeller plate. At voltages of +3000 and +4000 V of the repeller plate, the MCP gain voltages were varied to −1400, −1600 and −1800 V (−1400 and −1600 V) for the residual gas mode (Kr gas mode). Figs. 8 ▸(*a*) and 8 ▸(*b*) show the averaged profile distributions of the XIBPM and PM2, and their displacements at pink-beam mode of 500 eV and the residual gas mode, respectively. Because the beam path itself was adjusted by using the M3 mirror, all the profiles showed a similar amplitude, and they were also normalized by the profile that had the maximum height. While the X-profile of the PM2 showed a consistent distribution, the XIBPM and Y-profile of the PM2 showed changes according to the position of the M3 mirror. Compared with mono-beam mode, the pulse-by-pulse analysis showed a similar linear correlation with one of the average of a group of every ten pulses. The displacement ratios were determined by fitting the distributions of peak positions between the XIBPM and PM2 using a linear function for each setting. The displacement ratios of pink-beam mode with energies of 500, 700, 900 and 1100 eV were demonstrated based on voltages settings for the repeller plate with a fixed MCP gain of −1600 V and the MCP gain with a fixed repeller plate at +3000 V of the XIBPM (see Fig. 9 ▸). The Kr gas mode yields consistent results across the overall photon energy, regardless of the voltage settings and the number of pulses used for analysis. However, in the residual gas mode, particularly at a beam energy of 1100 eV, there was only a slight improvement despite averaging all pulses. When the MCP gain was not high enough, the results at 900 eV were also poor. This outcome appeared to be due to an insufficient signal-to-noise ratio. Even though the beam intensity was strong because it was a pink beam, the higher energy resulted in a lower cross section. The displacement ratios obtained for each experimental setting were compared with the distance ratio from the M3 mirror to the XIBPM and PM2. The distance ratio, specifically 1.75, was assumed to be accurate. Subsequently, the relative percentage errors were calculated for all data sets in pink-beam mode. The results for an average of a group of every ten pulses are shown in Fig. 10 ▸. In the residual gas mode, the errors increased as the beam energy increased, and the errors for 1100 eV photon energy demonstrated values of above 10% in most data sets, and the errors were improved slightly after averaging the profiles of all the pulses. Therefore, it was considered limited to use only the residual gas since the entire energy could not be covered. Adding the Kr gas improved the signal-to-noise ratio, and therefore the errors were largely improved at the data sets of 700, 900 and 1100 eV for the Kr gas mode. At 500 eV, a contradictory result was observed. However, the error was less than 6%. The relative errors for the average of a group of every ten pulses were estimated to be below 8% with the addition of Kr gas across the overall photon energy.

#### 3.2.3. Linearity

The four-blade slit scan data in mono-beam mode showed moderate errors with respect to the displacement ratios. However, the linearity of the XIBPM could be investigated. The amplitude of the XIBPM profile was compared with that of one of the PM1 X-profiles. For mono-beams with energies of 700 and 900 eV, the PM1 showed a saturation feature. The energy of 900 eV is the nominal energy to which the accelerator machine can be tuned. Therefore, among all the available energies at the soft X-ray beamline, 900 eV exhibited the highest photon flux. The PM detected the entire beam directly. Therefore, saturation is possible in reality without attenuators. For mono-beams with energies of 500 and 1100 eV, a linear correlation was observed between the XIBPM and the PM1. The *R*^2^ values obtained through a linear fitting were above 0.97 and 0.99 for the pulse-by-pulse and the average of a group of every ten pulses, respectively.

**Figure 11:**
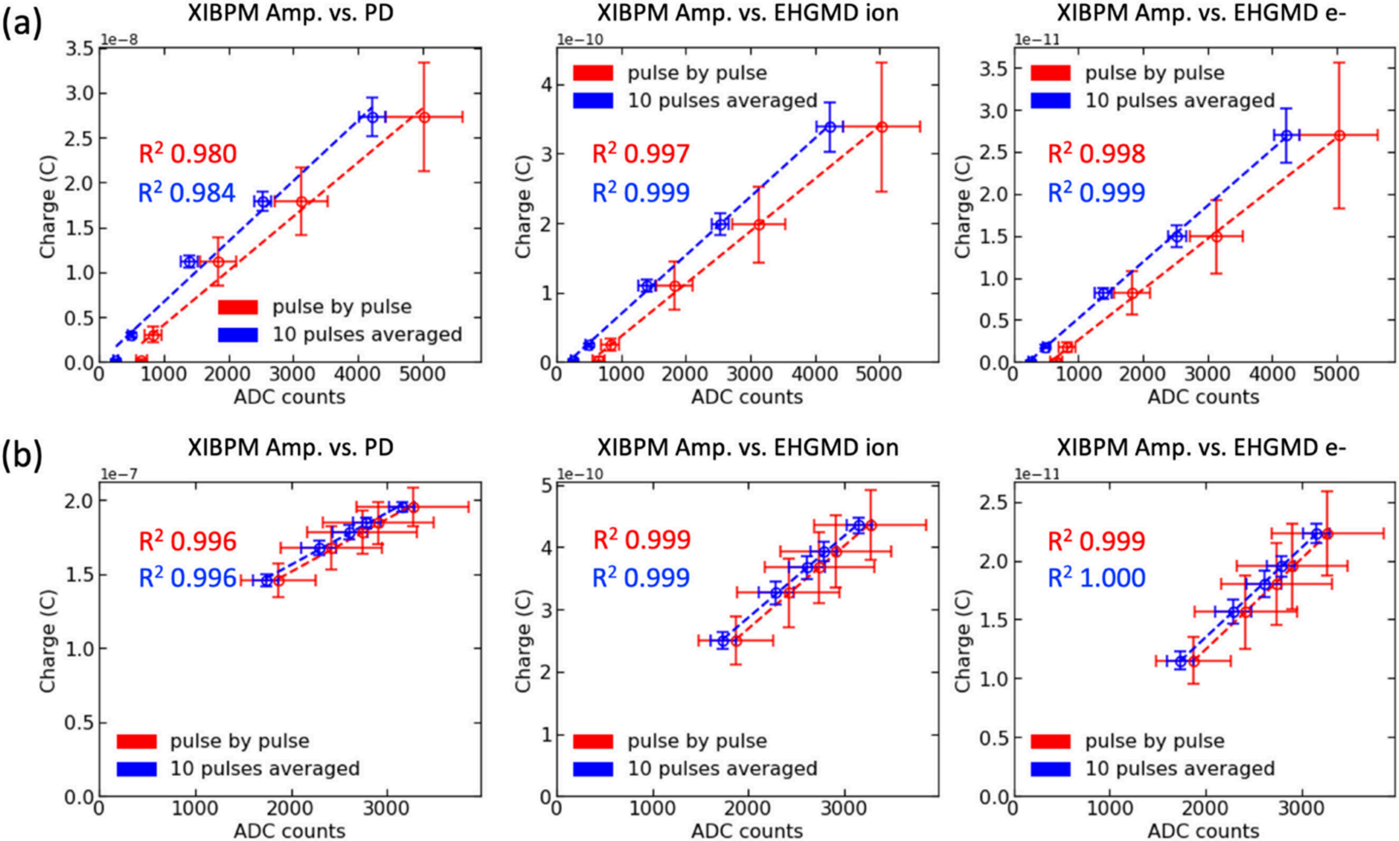
Correlations between the XIBPM amplitude and the charges measured by the PD, the EH-GMD ion and the EH-GMD e^−^ are shown for (*a*) mono-beam mode at 500 eV and (*b*) pink-beam mode at 1100 eV, both in the Kr gas mode.

To make a concrete confirmation regarding the linearity of the XIBPM, the data by attenuator scanning were obtained along with EH-GMD and PD within the test configuration shown in Fig. 3 ▸(*b*). The PD was located downstream of PM2, and detected the beam directly. The aluminium attenuators were set to five different thicknesses, and 300 X-ray pulses were acquired for each attenuator setting. Among the XIBPM voltage settings, the voltage for the repeller plate was fixed at +3000 V, while the MCP gain voltages were mainly adjusted to be −1400, −1600 and −1800 V. In the residual gas and mono-beam modes, if the MCP gain was not high enough, the amplitude changes of the XIBPM could not be easily observed depending on the attenuator thickness, even for an energy of 500 eV. After adding the Kr gas, the linearity could be clearly obtained in mono-beam mode except for the energy of 1100 eV. The correlations in pulse-by-pulse analysis (every ten pulses grouped) between the XIBPM amplitude and the EH-GMD e^−^ charge were obtained to be 0.96, 0.97, 0.97 and 0.10 (0.99, 0.99, 0.99 and 0.51) for 500, 700, 900 and 1100 eV, respectively, at the MCP gain voltage of −1600 V. In the residual gas and pink-beam modes, with an increase in the MCP gain, the beam energy at which the XIBPM showed linearity was extended. As a result, the *R*^2^ value was approximately 1 at 500, 700 and 900 eV, and 0.88 at 1100 eV for an average of a group of every ten pulses. However, in the Kr gas mode of pink-beam mode, the XIBPM showed a saturation feature at 500 eV even though the MCP gain voltage was set to be −1400 V, and it showed linearity at the other energies. With an increase in the MCP gain voltage, the saturation feature was revealed up to 900 eV, and subsequently the correlation distribution was well fitted by a quadratic function rather than a linear function. At 1100 eV, the XIBPM amplitude and EH-GMD e^−^ charge showed a correlation, regardless of the MCP gain voltages. Fig. 11 ▸ shows the correlation plots that were observed for mono-beam of 500 eV and pink-beam of 1100 eV in the Kr gas mode. The XIBPM amplitudes were compared with the charges of the PD, EH-GMD ion and EH-GMD e^−^, and showed good correlations.

## 4. Conclusions

The XIBPM for *in situ* beam position monitoring at the soft X-ray beamline of PAL-XFEL has been developed, utilizing photo-ionization of either residual gas or injected Kr gas. The trajectory of X-ray pulses was clearly visualized using the phosphor MCP and CCD camera of the XIBPM. The XIBPM was tested under various conditions, including the mono-beam and pink-beam modes, with energies of 500, 700, 900 and 1100 eV. Testing involved either residual gas in an ultra-high vacuum or injected Kr gas, as well as adjustments to the voltages of the repeller plate, MCP gain and grid to vary the electric field.

The basic behaviors of the profiles, such as amplitude, width and peak position, were evaluated based on beam energy and electric field. The amplitude increased significantly depending on the gain of the MCP, while it was less affected by increases in the voltage of the repeller plate. The impact of the electric field on profile width was particularly pronounced when using residual gas, resulting in widths measured to be six to nine times larger than the actual vertical beam size. Unexpectedly, peak position was also influenced by the electric field. The differences of the kinetic energy of the residual gas ions and Kr ions, as well as the electric field disturbance, were thought to be contributing factors.

Artificially altering the beam position and comparing the displacements between the XIBPM and PM confirmed the feasibility of monitoring relative changes in beam position. The addition of Kr gas across all photon energies resulted in relative percentage errors of below 8%. Achieving good linearity of 0.9 or higher required optimal conditions for a sufficiently large signal-to-noise ratio while avoiding saturation.

Simulations are underway to understand the unexpected effects of the electric field on broader profile width and peak position, as well as to find solutions to overcome the disturbing electric field. Improvement and optimization of the XIBPM can be realized by considering simulation results and enhancing the operation of pulse-by-pulse measurements.
